# Prevalence of human papillomavirus in saliva of women with HPV genital lesions

**DOI:** 10.1186/s13027-016-0096-3

**Published:** 2016-08-26

**Authors:** Giuseppa Visalli, Monica Currò, Alessio Facciolà, Romana Riso, Placido Mondello, Pasqualina Laganà, Angela Di Pietro, Isa Picerno, Pasquale Spataro

**Affiliations:** 1 Department of Biomedical and Dental Sciences and Morphofunctional Imaging, University of Messina, Via C. Valeria, Gazzi, 98100 Messina, Italy; 2 Riuniti Papardo Piemonte Hospital, Messina, Italy

**Keywords:** HPV, HSIL, Saliva, Head-neck cancer

## Abstract

**Background:**

The human papilloma viruses (HPVs) are DNA viruses associated with benign and malignant lesions of skin and mucous membranes. The HPVs has been implicated as the cause of virtually all cervical cancers worldwide but studies showed that these viruses can cause numerous cancers in several tissues including Oral Squamous Cell Carcinoma (OSCC). At least 90 % of HPV-positive OSCCs are associated with high-risk (or oncogenic) HPV-16 and oral infection confers an approximate 50-fold increase in risk for HPV-positive OSCC. HPV-positive OSCCs are associated with sexual behaviors in contrast to HPV-negative OSCCs that are associated with chronic tobacco and alcohol use. The aim of this study was to estimate the prevalence of HPV-DNA in saliva samples collected from women in which it has been previously established the HPV infection of the cervix with relative genotyping and, then, to study the possible correlation.

**Methods:**

Saliva samples were collected from 100 women with HPV cervical lesions, aged between 22 and 52 years old, and 25 healthy women with normal cytology (control group), aged between 20 and 49 years old. PCR assay was used to detect HPV DNA.

**Results:**

The prevalence of oral HPV infection in saliva samples was 24 % in women with HPV cervical lesions while in the control group was 8 %. It has been demonstrated a strong association between high grade squamous intraepithelial lesion and oral infection due to HPV16 and 18, that are the most frequently detected HPV genotypes.

**Conclusion:**

This study shows that patients with genital HPV infection are at risk for oral infection and, consequently, for the development of OSCC.

## Background

The human papilloma viruses (HPVs) are DNA viruses that infect squamous epithelial cells. They constitute a group of more than 100 different genotypes associated with benign and malignant lesions of skin and mucous membranes. These viruses are divided in two groups on the basis of their epidemiological association with the development of cervical carcinoma: high-risk HPVs, that include the genotypes 16, 18, 31, 33, 35, 39, 45, 51, 52, 56, 58, 59, 66 and 68 and low-risk HPVs such as the genotypes 6, 11, 42, 43 and 44. The HPV-DNA contains sequences that encodes two proteins with oncogenic capacity, E6 and E7, that exhibit their effect by disrupting the function of two tumor suppressor genes, p53 and pRb respectively, causing defective cell apoptosis and uncontrolled cell growth [1]. The HPVs has been implicated as the cause of virtually all cervical cancers worldwide [2] although they can infect several cell types causing various intraepithelial neoplasias [3–6]. Oral cancer holds the eighth position in the cancer incidence ranking worldwide [7]. Of all head and neck cancers, Oral Squamous Cell Carcinoma (OSCC) is the most common malignant epithelial neoplasia of oral cavity (90 %); this represent approximately 5 % in men and 2 % in women considering all malignancies [8]. At least 90 % of HPV-positive OSCCs are associated with high-risk (or oncogenic) HPV-16 [9] and oral infection confers an approximate 50-fold increase in risk for HPV-positive OSCC [3]. The incidence of OSCC has significantly increased over the last 3 decades in several countries, in particular, the incidence of HPV-positive OSCC increased by 225 % (from 0.8 per 100 000 to 2.6 per 100 000), predominantly among young individuals, and white men [10]. HPV-positive OSCCs are associated with sexual behavior in contrast to HPV-negative OSCCs that are associated with chronic tobacco and alcohol use [3]. Recently, besides the traditional risk factors for developing oropharyngeal cancer (tobacco use and heavy alcohol consumption), HPVs are identified as an independent risk factor in the onset of pre-cancerous and cancerous oropharyngeal lesions. It is likely, in fact, that HPV may modulate the malignancy process in some tobacco- and alcohol-induced oropharyngeal cancers, but may also be the primary oncogenic factor for inducing carcinogenesis in a subset of patients without these traditional risk factors [3, 4]. Of all the HPV genotypes, 24 are involved in the development of benign and malignant lesions of the oral cavity [11, 12]. In particular, HPV16, and to a lesser extent HPV18, are most commonly identified from oral biopsies [13, 14]. International studies have evaluated HPV prevalence in healthy adults using biopsy samples, revealing prevalence rates that ranged from 0 to 15 % [15, 16] and in healthy adult saliva and oral lavage samples, revealing prevalence rates between 2.8 and 25 % [13, 17, 18]. A recent systematic review of the literature showed oral HPV16 prevalence was 1.3 % among healthy individuals and appeared to differ by geographic region, although significant heterogeneity between studies due to in part to differences in specimen collection, processing and testing limited conclusive interpretation of the data [19]. There is limited information about the natural history of oral HPV infection, but since oral HPV16 infection is associated with this cancer, it is important to estimate the proportion of healthy individuals with oral HPV infection [19]. The aim of this study was to estimate the prevalence of HPV-DNA in saliva samples collected from women in which it has been previously established the HPV infection of the cervix with relative genotyping and, then, to study the possible correlation.

## Methods

### Patients

This study enrolled 100 women with HPV genital lesions and 25 healthy women (control group), selected from a group of 280 women that went to the Gynaecological Unit of the Riuniti Papardo-Piemonte Hospital, in Messina, for routine gynaecological screening between July 2014-October 2015. The control group was selected on the base of negative Pap-Test from last 3 years. None of the women enrolled in the study was vaccinated for HPV. The gynaecologist, after making sure of the absence of lesions in the oral cavity, had provided to us a saliva sample and all the clinical information of patients.

After having received their written informed consent, a detailed anonymous questionnaire was administered in order to collect information on age, gender, smoking, drinking and lifetime sexual activity.

### Sample collection and DNA isolation

Participants were asked to sit comfortably in an upright position and tilt their heads down slightly to pool saliva in the mouth.

The first expectoration was discarded to eliminate food debris and unwanted substances contaminating the sample that may cause analytical inaccuracy.

Participants were asked, just after, to briefly (for 30 s) refrain from swallowing and expectorate however much saliva was in the mouth from a single expectoration into a pre-labelled sterile container and ~ 2 mL saliva was collected. The samples were then immediately refrigerated to minimize degradation of salivary proteins until further processing. To process, the oral rinse was centrifuged at 3,000 g for 10 min at 4 °C, the supernatant was removed and the pellet was resuspended in 10 ml of sterile normal saline; the centrifugation was repeated and the salivary pellet was stored at -80 °C until DNA purification.

### DNA extraction

DNA was extracted from saliva cellular pellets using Puregene DNA purification system (Qiagen, Milan, Italy) according to manufacturer’s instructions. DNA concentration and quality were estimated by spectrophotometer measurements of absorbance at 260 and 280 nm and electrophoresis.

### Detection of HPV in the saliva sample

Total saliva DNA was tested for HPV DNA by a PCR assay using the consensus primers MY09/MY11, which amplify a fragment of 450 bp within the L1 gene region of the viral genome (Table 1).

**Table 1:** List of primers and annealing temperatures used in this study

Primer name	Sequence (5’ → 3’)	Annealing temperature (°C)	Fragment lenght (bp)
MY09 CGTCCMARRGGAWACTGATC		58	450
MY11 GCMCAGGGWCATAAYAATGG			
HPV6f TAGTGGGCCTATGGCTCGTC		55	280
HPV6r TCCATTAGCCTCCACGGGTG			
HPV11f GGAATACATGCGCCATGTGG		58	360
HPV11r CGAGCAGACGTCCGTCCTCG			
HPV16f TGCTAGTGCTTATGCAGCAA		55	152
HPV16r ATTTACTGCAACATTGGTAC			
HPV18f AAGGATGCTGCACCGGCTGA		58	216
HPV18r CACGCACACGCTTGGCAGGT			
HPV31f ATGGTGATGTACACAACACC		55	514
HPV31r GTAGTTGCAGGACAACTGAC			
HPV33f ATGATAGATGATGTAACGCC		55	455
HPV33r GCACACTCCATGCGTATCAG			
HPV45f ATTTCACAGCATAGCTGGACAGTA		55	100
HPV45r CTATACTTGTGTTTCACTACGTCT			
ß-globin f GAAGAGCCAAGGACAGGTAC		57	268
ß-globin r CAACTTCATCCACGTTACC			

Each PCR run included a negative (sterile water substituted for DNA) and a positive (DNA sample of an HPV type 16 carrier) control to monitor contamination and overall end point sensitivity.

In parallel, each sample was amplified for β-globin to control for DNA integrity.

PCR reactions were carried out in a total volume of 50 μl containing purified DNA (200 ng), 1x PCR Buffer, 3 mM MgCl_2_, 1 U of Taq DNA polymerase, 0.2 mM of dNTP, and 0.3 μM of each primer for HPV genome (MY09/MY11) or β -globin.

Amplification was performed in a Hybaid PCR sprint thermocycler with the following profile: an initial denaturation step at 94 °C for 10 min, followed by 40 cycles of denaturation at 94 °C for 1 min, primer annealing at 58 °C for 1 min, and extension at 72 °C for 1 min; finally, an extension step of 7 min at 72 °C.

The PCR products were analyzed by 2 % agarose gel electrophoresis, stained with ethidium bromide and visualized with ultraviolet transilluminator.

Then, HPV-positive samples were amplified with a set of seven different-HPV-type specific primers. The sequences of primers and annealing temperatures used are given in Table 1.

### Statistical analyses

The association between HPV infection and socio-clinical variables was assessed using chi-square tests, evaluating the Odds Ratio (OR) and the 95 % Confidence Interval (CI). Significance was assessed at the *p* < 0.05 level. All analyses were performed using Prism 4.0 software.

## Results

Table 2 shows information on age, gender, smoking, drinking, dietary and sexual habits obtained by anonymous questionnaire.

**Table 2:** Behavioural factors of studied subjects

Variable	HPV negative	HPV positive
Age media at first sexual intercourse	20	17
Sexual promiscuity		
*Yes*	0	9
*No*	25	91
Oral sex habit		
*Yes*	3	15
*No*	22	85
Use of sex-toys		
*Yes*	0	2
*No*	25	98
Smoking habit		
*Yes*	7	38
*No*	18	62
Alcohol consumption		
*Yes*	8	40
*No*	17	60
HPV vaccine		
*Yes*	0	0
*No*	25	100
Coinfections		
*Yes*	0	23
*No*	25	77

Saliva samples were collected from 100 women with HPV cervical lesions, aged between 22 and 52 years old, and 25 healthy women with normal cytology (control group), aged between 20 and 49 years old.

In particular, in women with HPV cervical lesions, according to the Bethesda System Cytology Classification, on the basis of lesions, condylomas accounted for 47 % of the women, low-grade squamous intra-epithelial lesions (LSILs) for 28 % and high-grade squamous intra-epithelial lesions (HSILs) for 25 %.

The information on cervical HPV genotyping supplied by gynaecologist showed that 42 samples were positive for low-risk genotypes, and the remaining 58 samples were positive for high-risk genotypes. Of all HPV cervical samples, 25 were positive for more than one HPV type.

Considering single and multiple infections, HPV 16 was the most frequent type (19 samples), followed by types 6 (13 samples), 45 and 11 (8 samples each), 81 (6 samples), 18, 52 and 31 (4 samples each).

The concentration of DNA isolated from all collected saliva samples was between 100 and 300 ng/μL. Absorbance measurements and A260/A280 ratio analysis confirmed the purity of the DNA isolates, which averaged between 1.7 and 2.0.

The results of PCR with MY09/MY11 primers showed that the HPV positive saliva samples were 24 in women with HPV cervical lesions and 2 in the control group.

**Fig. 1:**
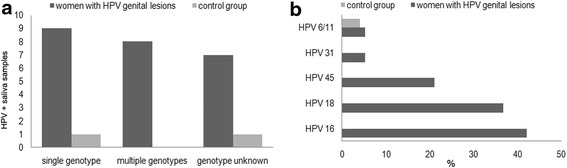
**a** Women infected by multiple genotypes simultaneously, one genotype and unknown genotype in saliva samples. **b** Genotype characterization of most frequent HPVs in saliva of women with HPV genital lesions and in control group

Of these positive samples, the characterization of HPV genotype showed that, in women with HPV cervical lesions, 9 woman had a multiple infection (more genotypes simultaneously), 8 women were infected with one genotype while 7 women were positive for MY09/MY11 but no specific genotype between those tested was detected. In the control group, 1 woman was infected with one genotype and 1 woman was positive for MY09/MY11 but no specific genotype between those tested was detected (Fig. 1a).

The Fig. 1b shows the genotype characterization, HPV 16 was the most frequent followed by the 18 and then from 45. The percentage of positivity to low-risk HPV infection (caused by HPV 6/11) was about 5.26 %.

To investigate in the HPV positive cervical group a potential risk factor promoting the presence of the virus in the saliva we correlated this data with the information collected in the questionnaire.

There was no statistical significance of association between the HPV positivity and patients’ age, smoking and drinking alcohol.

Other social and sexual behaviors were not significantly associated with the detection of HPV DNA in saliva samples.

**Fig. 2:**
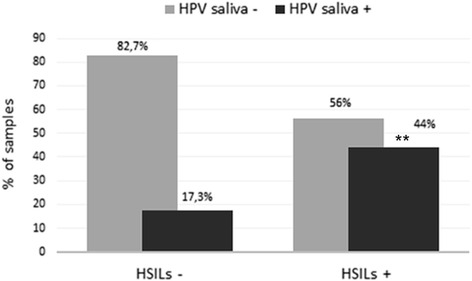
Comparison of the HPV positivity frequency in oral fluid between HSIL positive and negative groups

Analyzing the data of saliva HPV positivity and cervical clinical data we revealed a correspondence of genotypes between saliva and cervix in case of infections by HPV 16 and 18 supported by a significant increase of saliva HPV positivity in women with high-grade cervical lesions (*P* = 0.0069; OR = 3.747; 95 % CI: 1.39–10.09) (Fig. 2).

## Discussion

The diagnosis of cervical HPV infections generally is not accompanied by investigations in different sites such as the oral cavity, except in the presence of visible lesions. Our results show that patients with genital HPV infection are at risk for oral infection not always associated with injuries. The absence of clinical signs in the oral cavity of these patients suggests a subclinical infection, and a molecular assay might thus be necessary to diagnose it.

Highly conserved regions in different parts of the viral genome have enabled the development of general or consensus PCR primer sets which allow the detection of a broad spectrum of different HPV genotypes. However, differences in malignant potential mean that it is particularly important to accurately identify infections with the high-risk HPV genotypes. After amplification with general or consensus primers, additional techniques are necessary to identify the underlying HPV genotype [20, 21].

In the present study, we performed a careful oral clinical examination of all patients; no injury was found. We chose to investigate women with cervical HPV but no visible lesions in the oral cavity in order to demonstrate the presence of the virus in saliva even in the absence of evident clinical signs. This finding indicates that oral examination alone can not exclude the possibility of oral HPV infection.

A link between human papillomavirus and oropharyngeal cancer was suggested more than 20 years ago [22]. Recent studies of healthy children and adults have found an oral prevalence of high-risk HPV strains, ranging between 2.5 and 5 % [17, 19] while previous studies revealed oral-HPV in 20.7 % of women with concomitant HPV-cervical lesions [13, 23]. Our study highlights that women with a prior histopathologic diagnosis of cervical HPV are at high risk for subclinical oral HPV, as indicated by the presence of the virus in the oral cavity of 24 % of the patients. In particular, 70 % were positive to high-risk (HPV 16-18-31-33-45) and low-risk (6–11) most frequent HPV genotypes. A significant proportion (30 %) of subjects has not been characterized; we assume that the positivity could be due to infection with other less frequent genotypes. Despite the wide distribution of HPV in general population, we found a low positivity in the control used in this study.

Previous studies have shown that current smoking (and intensity) is associated with oral HPV infection [24, 25]. Actually, even if we did not obtain a significative statistical correlation between smoking habit and oral infection, probably because the studied women were occasional smokers, it cannot be excluded a possible synergy between these two factors. This hypothesis is suggested by the well known oxidative damage caused by smoke that is an important risk factor for the HPV infection as previously shown [26].

Moreover, oral sexual behaviors have been associated with oral HPV infection and transmission of other viral infections, such as herpesviruses (HSV) [27]. The majority of the women examinated in our study stated that don’t have oral sexual habits but we think that this is a false finding, due to the social conditioning; this is suggested by the correlation between cervical and oral genotypes found in our study. A multicenter study revealed a percentage of the HPV detection in oral cancer specimens higher among subjects who have oral sexual habits and/or sexual promiscuity [13].

## Conclusion

The transmission in the oral cavity of HPV and, consequently, the risk of oral cancer is increased in women with cervical cancer and in their spouses [13, 28]; this finding suggests a cross-transmission between the oral cavity and genitals. Our results, in accordance with the cited studies, highlight the need to perform an oral screening test in the women with cervical high risk-HPV lesions. The correlation between these two anatomic sites could be consequent to genetic predisposition and/or conditions of low immune response such as HIV infection [29, 30] that probably favor the colonization and the persistence of oral HPV [31].

To prevent the cross-transmission, it could be useful focusing the attention on a correct health education to reduce the risk of oral and, in general, head and neck cancer developing. It is important, in our opinion, increase not only correct sexual behaviors but, in general, healthy lifestyle.

The US Centers for Disease Control and Prevention (CDC) currently recommends routine HPV vaccination for females aged 9 to 26 years and males aged 9 to 21 years for the prevention of ano-genital warts and cancers based on demonstrated efficacy in randomized clinical trials [32, 33]. Current vaccines approved by the FDA prevent infections with HPV types 16 and 18, the two high-risk HPVs that cause about 70% of cervical cancers and the most part of the other HPV-associated cancers [10, 34].

Even if the vaccine efficacy against oral HPV infection is unknown, and therefore the vaccination cannot currently be recommended for the primary prevention of oropharyngeal cancer, this practice could be equally useful in the prevention of this kind of cancer considering that the most detected oral HPV genotypes, in our study, were HPV-16 and 18 and the strong demonstrated correlation between HSIL and oral HPV positivity.

## Abbreviations

HPV, human papillomavirus; OSCC, oral squamous cell carcinoma; HSIL, high grade squamous intraepithelial lesion; LSIL, low grade squamous intraepithelial lesion; HSV, herpesvirus; CDC, center for disease control and prevention; FDA, Food and Drug Administration
